# Transmission of Light through Animal Structure

**Published:** 1872-12

**Authors:** 


### ARTICLE X.

### *Transmission of Light through Animal Structure.*

A statement copied from the *San Francisco Scientific Press* is now going the round of the newspapers, to the effect that a method has recently been discovered in America, by which the internal organs of the body can be rendered visible. This statement requires to be corrected, in order that a true version of the known facts, on the subject referred to, may be presented to the public.

The report states that Dr. Richardson, two years ago, accidentally discovered, in conducting some electrical experiments, that the hand can be so illuminated by the electric light as to be rendered transparent, and that Dr. Thomas Nicholson, of New Orleans, pursuing the inquiry further, has, by aid of the calcium light shown it possible to bring the internal organs of the body into view for diagnostic purposes. The facts really are, that Dr. Richardson's experimental observations were in no way accidental, neither were they confined to the application of the electric light. On the contrary, they were parts of a long, laborious, and systematic research conducted five years ago, and reported to the British Association for the Advancement of Science at its meeting at Norwich in 1868. In his researches Dr. Richardson employed, in various ways, the electric light, the magnesium light, and the calcium light; and at Norwich, where he explained the relatlve values of these different illuminating agencies, the instrument he exhibited was worked with the magnesium light. His inquiries included the study of the effects of light on portions of the dead body, on bone, muscle blood, membrane and parts of visceral organs; while they also included attempts to make visible the internal structures of certain regions of the living body—viz., the hand and foot, the structures forming the cheeks, the reck, the chest, and the abdomen. Since that time the same investigator has pursued the inquiry, thus independently originated, and has a few times successfully applied the process in diagnosis.

As a summary of what has yet been ascertained, we may state that the absorption of light by the blood, and the general diffusion of blood through the living organism, seriously and immediately limit success, even in parts most diaphanous. In structures the least dense, the pulsations of the arterial vessels—i. e., the motions of pulsation—may be seen, though the vessels themselves are not rendered distinct; nerves are not defined, and bony structures are but obscurely outlined. In an extremely emaciated young subject, when the magnesium light was, with a suitable apparatus, directed upon the thorax laterally, on the left side, the form of the heart was faintly discernable, but the alternate motions of the auricles and ventricles could not be distinguished. The presence of cysts containing fluids, of accumulations of fluids in serous cavities, and of well-defined dense internal tumors, might, Dr. Richardson thought, be detected in cases favorable for this mode of examination, and in the diagnosis of some obscure forms of hernia the method might possibly be applied with advantage.

Some observations have been made by the same inquirer, on the transmission of light through some of the inferior nimals, such as the frog, the chick, and the cary.

The difficulties that have stood in the way of a satisfactory practical application of this intended advance in medical science and art are very numerous. We have pointed out certain of these, but there are others connected with the working of the instruments required, and with the absorption of heat, all of which difficulties have yet to be overcome. These have prevented Dr. Richardson publishing the results of his labors since 1868, his wish being to present a method so complete and practical that all medical men may at once avail themselves of it for diagnosis. The recent announcement from the other side of the Atlantic may, perhaps, lead him to bring before the profession his later efforts towards the perfection of a process which may, one day in the future, form a subject of considerable historical interest in the annals of medicine.—*London Lancet.*

---

